# In Memory of Dr. W. B. Chambers

**Published:** 1898-03

**Authors:** 


### In Memory of Dr. W. B. Chambers.

At a meeting of the dentists of Newark city and county held at the office of Dr. R. A. Barrick, February 10th, 1898, at 7:00 P. M., the following resolutions were unanimously passed:

WHEREAS, It has pleased the Creator and final disposer of all things to remove from this world our brother, Dr. W. B. Chambers, of Newark city. Therefore be it

*Resolved,* That we bow to the will of Almighty God and yet desire to emphasize our grief in the death of our professional brother, and to bear testimony of his abilities as an operator and mechanic, which were of such high standard, having but few equals and no superiors; and the death of Dr. Chambers removes from the group of dental practitioners of Licking County, one who was universally respected and admired for his professional abilities by his brother dentists, as well as the public generally, and one whose place will be hard to fill.

*Resolved,* That we extend to his bereaved wife our sincere sympathy, and with reverent humility we commend her to Him who alone can all her sorrows heal.

*Resolved,* That as a token of respect we all close our offices at 12:00 o'clock NOON, on Saturday, and attend the funeral of our late brother.

*Resolved,* That a copy of these resolutions be sent to each of the daily papers and also to several of the dental journals and a copy be transmitted to the widow.

DR. MCCLEERY,<br>

DR. WOODS,<br>

DR. BARRICK,<br>

DR. SEDGWICK. } *Committee.*

